# Supplementary material for: A comparison of four epidemic waves of COVID-19 in Malawi; an observational cohort study
Source: BMC Infect Dis. 2023 Feb 7;23:79. doi: 10.1186/s12879-022-07941-y (PMC9902830; doi:10.1186/s12879-022-07941-y)
Supplement: Supplementary file 2 — Additional file 2. Supplementary figures. [file 12879_2022_7941_MOESM2_ESM.pptx]

## Slide 1
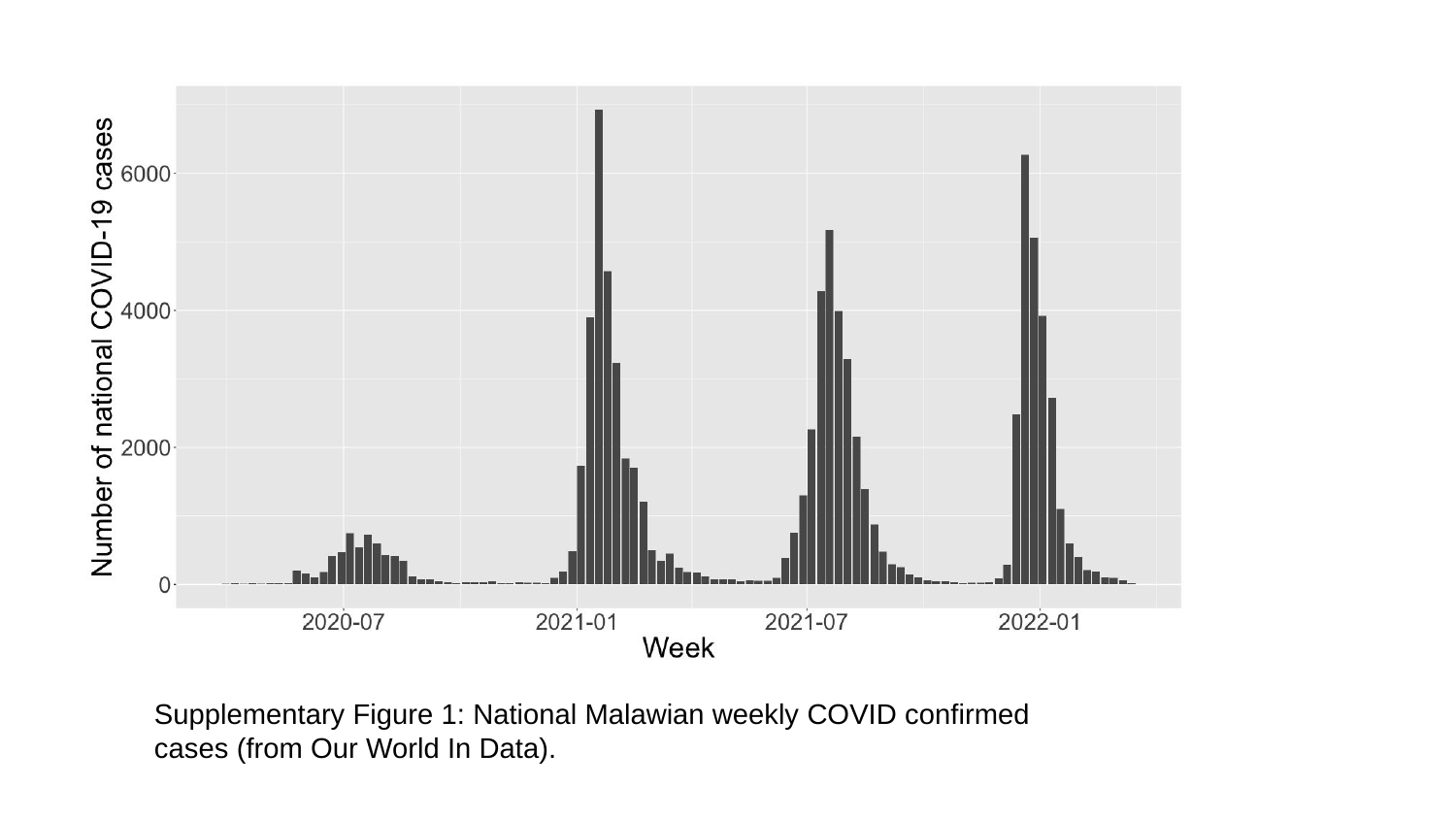

Supplementary Figure 1: National Malawian weekly COVID confirmed cases (from Our World In Data).

## Slide 2
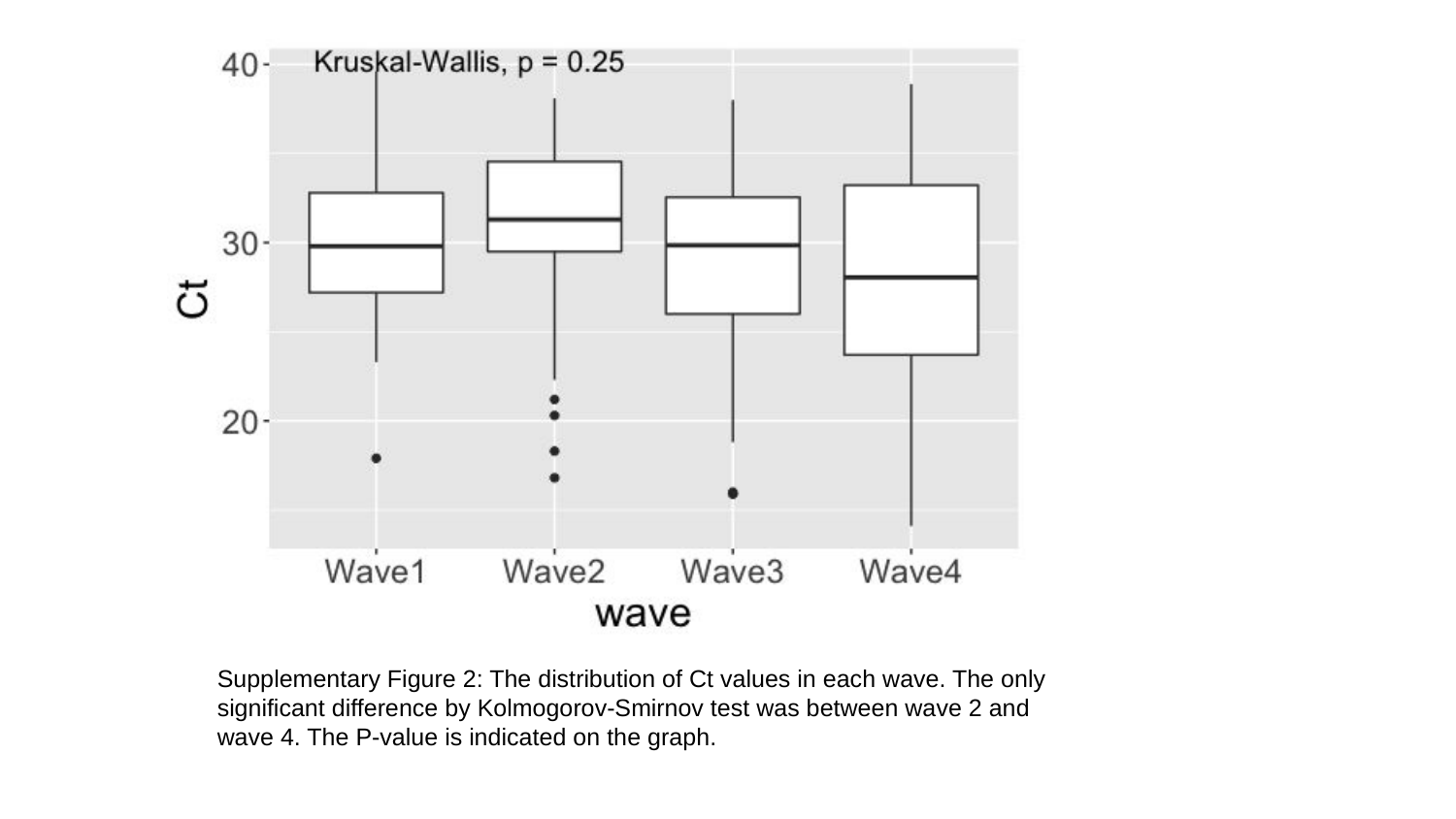

Supplementary Figure 2: The distribution of Ct values in each wave. The only significant difference by Kolmogorov-Smirnov test was between wave 2 and wave 4. The P-value is indicated on the graph.

## Slide 3
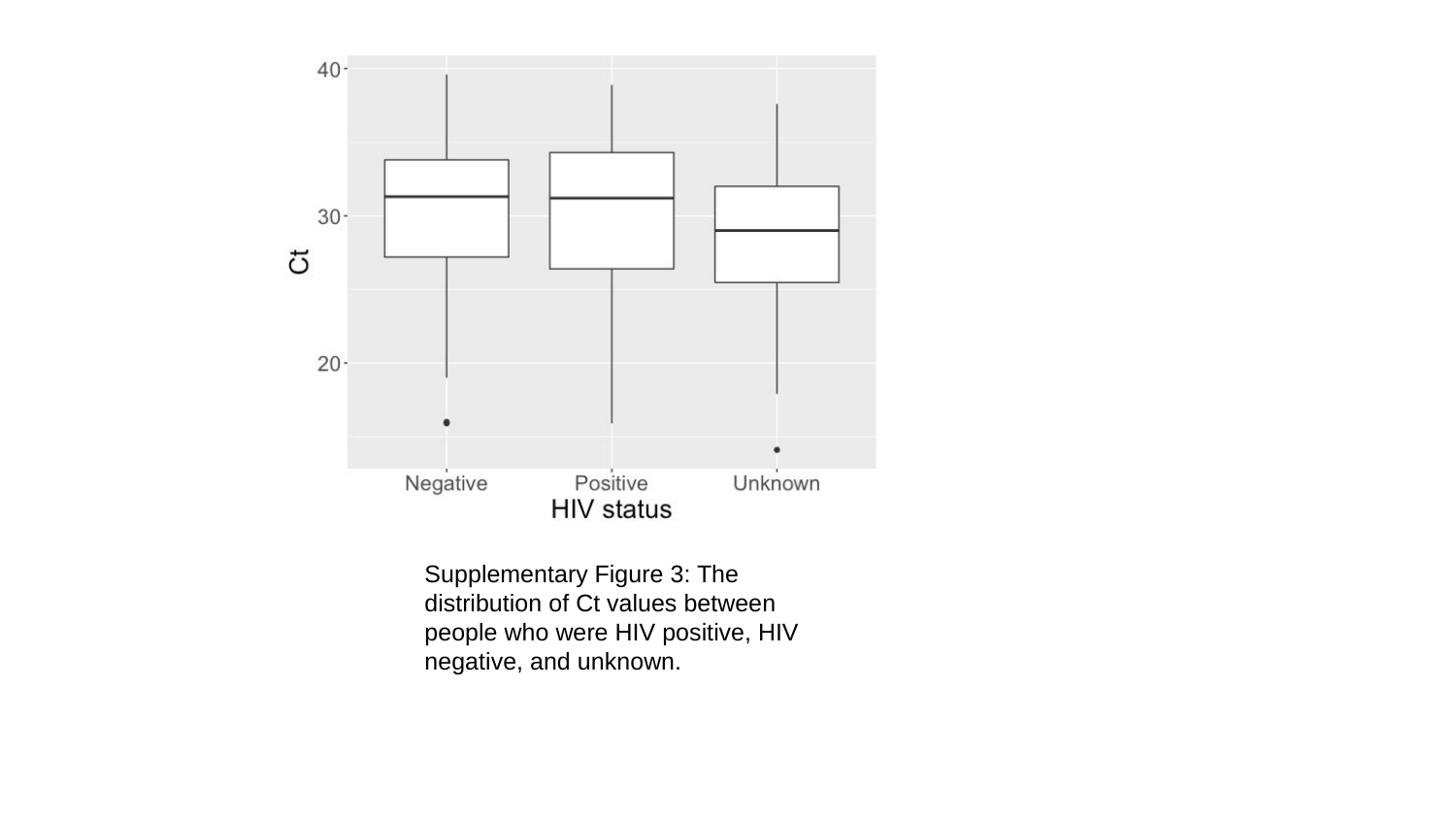

Supplementary Figure 3: The distribution of Ct values between people who were HIV positive, HIV negative, and unknown.

## Slide 4
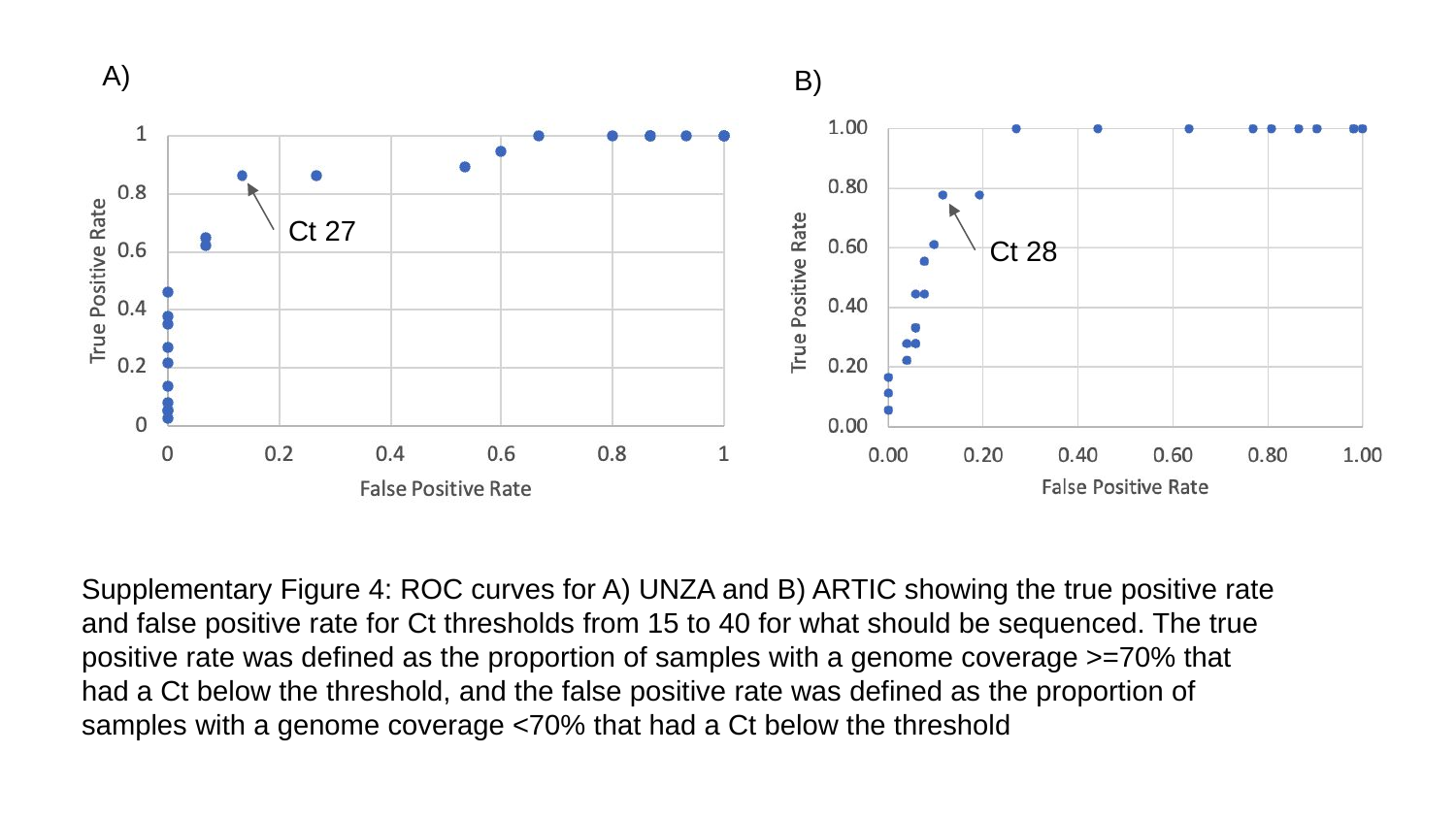

A)
B)
Ct 27
Ct 28
Supplementary Figure 4: ROC curves for A) UNZA and B) ARTIC showing the true positive rate and false positive rate for Ct thresholds from 15 to 40 for what should be sequenced. The true positive rate was defined as the proportion of samples with a genome coverage >=70% that had a Ct below the threshold, and the false positive rate was defined as the proportion of samples with a genome coverage <70% that had a Ct below the threshold

## Slide 5
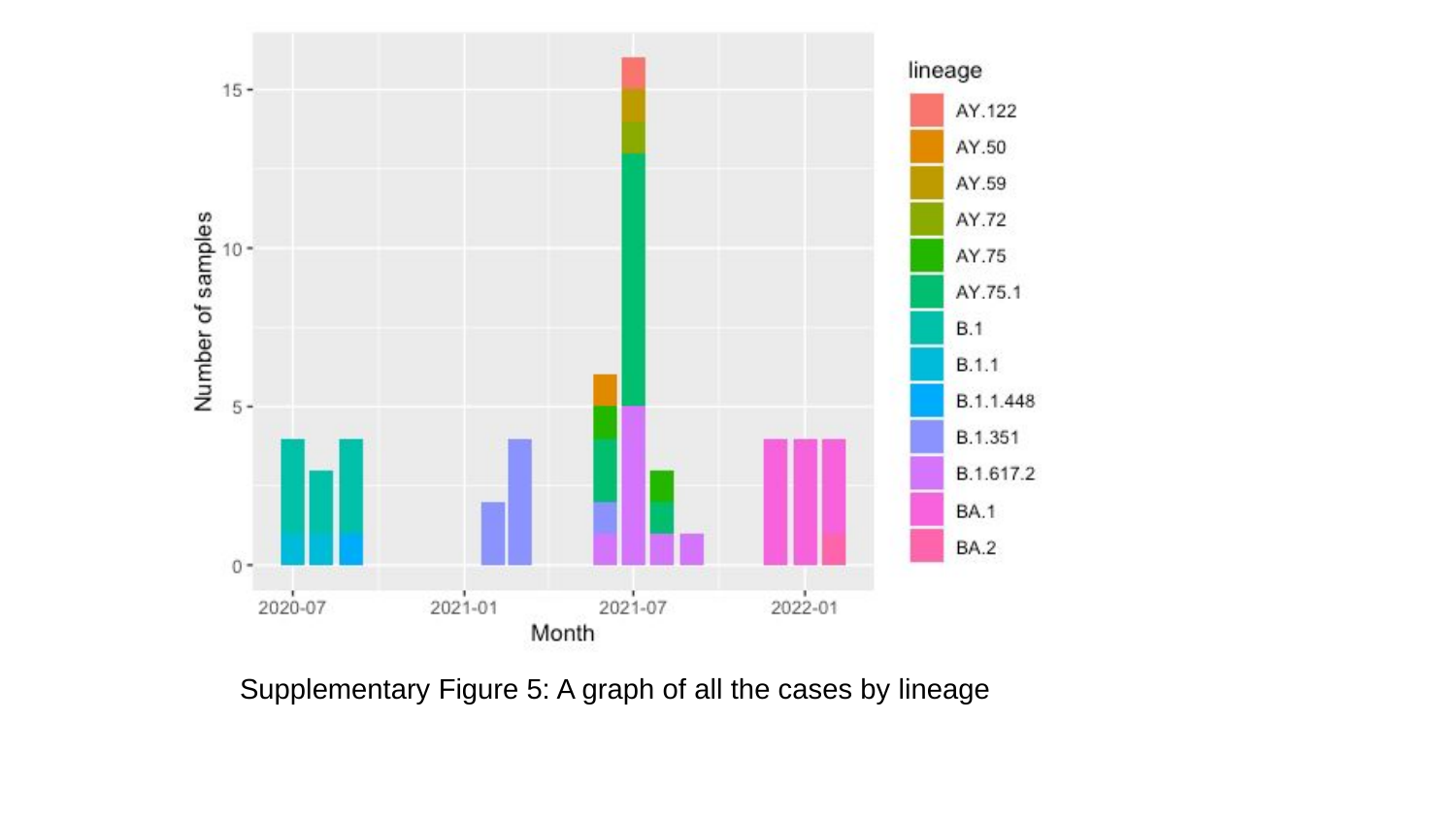

Supplementary Figure 5: A graph of all the cases by lineage

## Slide 6
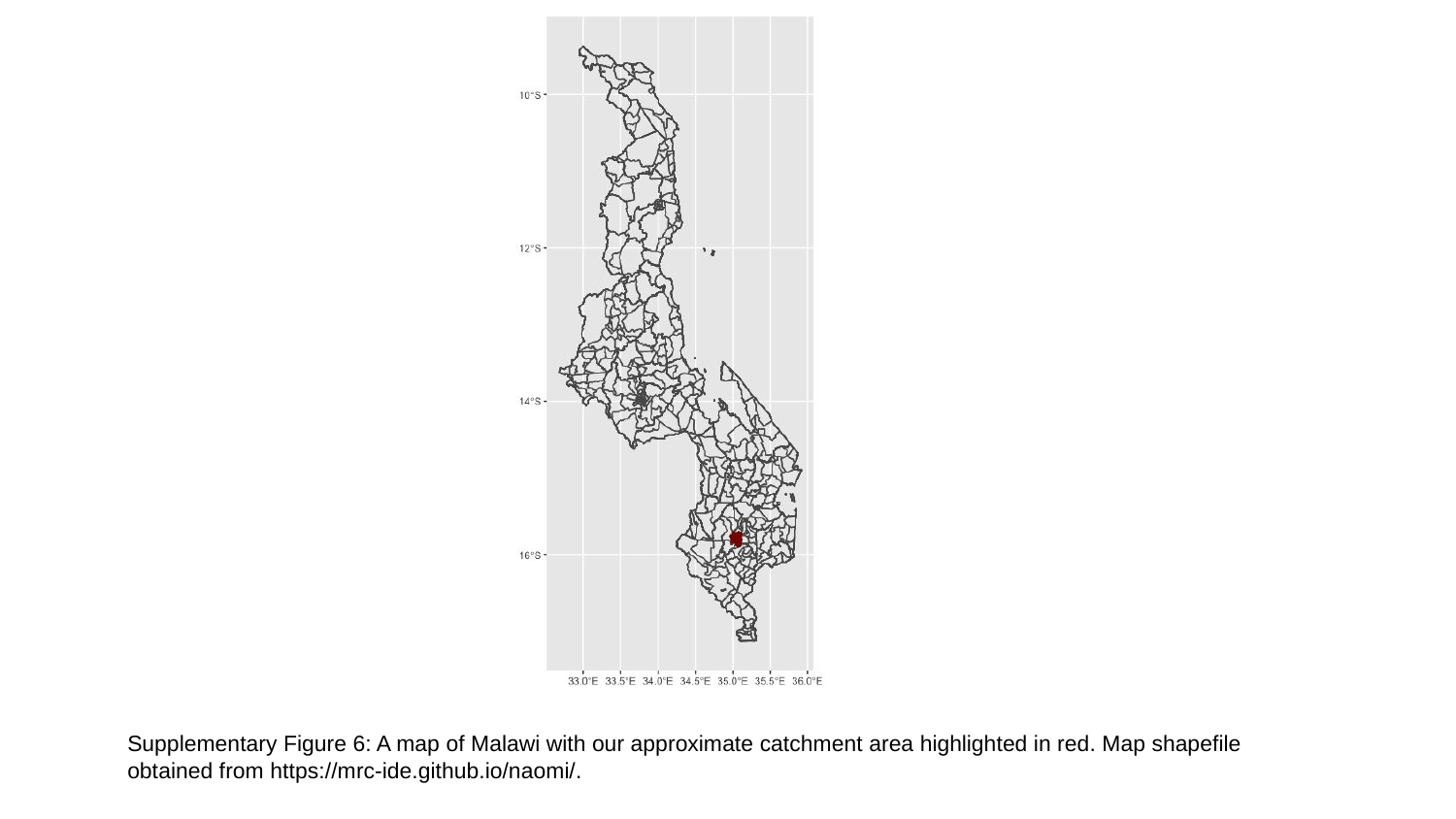

Supplementary Figure 6: A map of Malawi with our approximate catchment area highlighted in red. Map shapefile obtained from https://mrc-ide.github.io/naomi/.
